# Supplementary material for: Identification of photoperiod-induced specific miRNAs in the adrenal glands of Sunite sheep (Ovis aries)
Source: Front Vet Sci. 2022 Jul 22;9:888207. doi: 10.3389/fvets.2022.888207 (PMC9354845; doi:10.3389/fvets.2022.888207)
Supplement: Supplementary file 1 [file Data_Sheet_1.ZIP › Supplementary file/miRDeep2 file.zip!/miRDeep2 file/Novel_Structure/LP42A2/Novel_470.pdf]

Provisional ID	:	NC_019484.2_38238
Score total	:	57.3
Score for star read(s)	:	3.9
Score for read counts	:	50.1
Score for mfe	:	1.7
Score for randfold	:	1.6
Score for cons. seed	:	
Total read count	:	110
Mature read count	:	107
Loop read count	:	0
Star read count	:	3

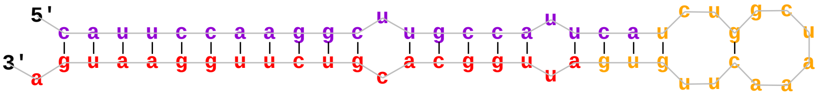

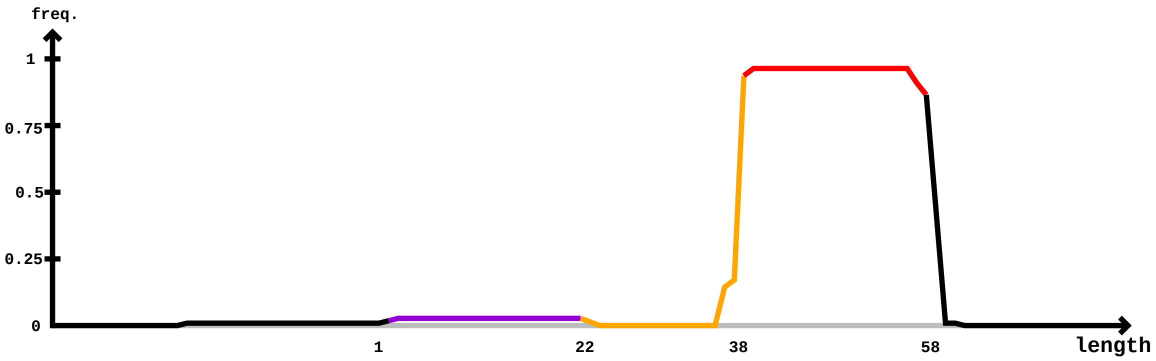


```
                                  Star                                 Mature
5'- uugccaccuucagcgugucugugugcuguaccccauuccaaggcuugccauucaucuggcuaaacuugugauuggcacgucuuggaaugaaguaacguacaacaaugaug   -3'   obs
    uugccaccuucagcgugucugugugcuguaccccauuccaaggcuugccauucaucuggcuaaacuugugauuggcacgucuuggaaugaaguaacguacaacaaugaug         exp
    ..((........))..((((((((((..(((..(((((((((((.(((((.((((..(......)..)))).))))).)))))))))))..)))..)))).)))..))).   reads    mm     sample
    ............gcgugucugugugcuguaccc.............................................................................   1        0      seq
    .................................cauuccaaggcuugccauuca........................................................   2        0      seq
    ..................................auuccaaggcuugccauuca........................................................   1        0      seq
    ....................................................................ugauuggcacgucuuggaau......................   4        0      seq
    ....................................................................ugauuggcacgucuuggaaug.....................   2        0      seq
    ....................................................................ugauuggcacgucuuggaauga....................   8        0      seq
    ....................................................................ugauuggcacgucuuggaaugaa...................   2        0      seq
    .....................................................................gauuggcacgucuuggaauga....................   3        0      seq
    ......................................................................auuggcacgucuuggaau......................   2        0      seq
    ......................................................................auuggcacgucuuggaaug.....................   3        0      seq
    ......................................................................auuggcacgucuuggaauga....................   58       0      seq
    ......................................................................auuggcacgucuuggaaugaa...................   22       0      seq
    .......................................................................uuggcacgucuuggaauga....................   1        0      seq
    .......................................................................uuggcacgucuuggaaugaa...................   1        0      seq
    .......................................................................uuggcacgucuuggaaugaagu.................   1        0      seq
```
